# The Effect of Inversion at 8p23 on *BLK* Association with Lupus in Caucasian Population

**DOI:** 10.1371/journal.pone.0115614

**Published:** 2014-12-29

**Authors:** Bahram Namjou, Yizhao Ni, Isaac T. W. Harley, Iouri Chepelev, Beth Cobb, Leah C. Kottyan, Patrick M. Gaffney, Joel M. Guthridge, Kenneth Kaufman, John B. Harley

**Affiliations:** 1 Center for Autoimmune Genomics and Etiology, Cincinnati Children's Hospital Medical Center (CCHMC), 3333 Burnet Avenue, Cincinnati, OH, 45229, United States of America; 2 University of Cincinnati, College of Medicine, Cincinnati, OH, United States of America; 3 Division of Biomedical Informatics, Cincinnati Children's Hospital Medical Center, Cincinnati, OH, 45229, United States of America; 4 Division of Rheumatology and Department of Internal Medicine, University of Colorado Denver, Aurora, CO, 80045, United States of America; 5 Arthritis and Clinical Immunology, Oklahoma Medical Research Foundation, Oklahoma City, Oklahoma, 73104, United States of America; 6 Department of Veteran Affairs Medical Center, Cincinnati, OH, United States of America; Oklahoma Medical Research Foundation, United States of America

## Abstract

To explore the potential influence of the polymorphic 8p23.1 inversion on known autoimmune susceptibility risk at or near *BLK* locus, we validated a new bioinformatics method that utilizes SNP data to enable accurate, high-throughput genotyping of the 8p23.1 inversion in a Caucasian population. Methods: Principal components analysis (PCA) was performed using markers inside the inversion territory followed by k-means cluster analyses on 7416 European derived and 267 HapMaP CEU and TSI samples. A logistic regression conditional analysis was performed. Results: Three subgroups have been identified; inversion homozygous, heterozygous and non-inversion homozygous. The status of inversion was further validated using HapMap samples that had previously undergone Fluorescence in situ hybridization (FISH) assays with a concordance rate of above 98%. Conditional analyses based on the status of inversion were performed. We found that overall association signals in the *BLK* region remain significant after controlling for inversion status. The proportion of lupus cases and controls (cases/controls) in each subgroup was determined to be 0.97 for the inverted homozygous group (1067 cases and 1095 controls), 1.12 for the inverted heterozygous group (1935 cases 1717 controls) and 1.36 for non-inverted subgroups (924 cases and 678 controls). After calculating the linkage disequilibrium between inversion status and lupus risk haplotype we found that the lupus risk haplotype tends to reside on non-inversion background. As a result, a new association effect between non-inversion status and lupus phenotype has been identified ((p = 8.18×10^−7^, OR = 1.18, 95%CI = 1.10–1.26). Conclusion: Our results demonstrate that both known lupus risk haplotype and inversion status act additively in the pathogenesis of lupus. Since inversion regulates expression of many genes in its territory, altered expression of other genes might also be involved in the development of lupus.

## Introduction

The inversion polymorphism at 8p23.1 is one of the largest variants found in man, encompassing 4.5 Mb [1], [2] with estimated frequencies that depend on population, 20%–50% in Europeans, 59% in the Yoruba and 12%–27% in Asians [3]–[5]. The fluorescence in situ hybridization (FISH)-based assay is often considered a gold standard to detect chromosomal inversion; however, it is not an effective method when a large number of samples are required to characterize inversions on a population-level. Recently a new statistical method based on principal components analysis (PCA) using unphased high-snp density genotyping data was successfully introduced [6]. The rational for this approach was built upon the concept of suppression of recombination events between two segments of different orientations in the inverted region [7]–[10]. Because of lack of recombination events, the two segments represent two distinct lineages that have been diverging for many generations and accumulating mutations independently. SNPs within the inverted region should therefore have different statistical properties, as if they were from different populations. This population substructure can be readily detected using PCA resulting in a special pattern in the distribution of samples in the space spanned by the first few eigenvectors. This special pattern, consisting of three equi-distant stripes is indicative of inversions and can be used to infer the inversion status of the samples [6]. Previous simulation studies suggest that as few as 150 markers in inverted regions is sufficient to generate a meaningful PCA analyses, however that depends on the type of markers used for the study and the level of linkage disequilibrium (LD) with inversion [6]. Although some candidate tag markers have been reported to determine inversion, the results were mostly inconsistent, mainly because there are no known SNPs in complete LD with inversion that can act as a perfect proxy for the inversion [11]. We are especially interested in the 8p23 inversion because the *FAM167A/BLK* locus is located inside this inverted segment. *BLK* (B Lymphoid Tyrosine Kinase) encodes a nonreceptor tyrosine-kinase of the src family and is involved in B-lymphocyte development, differentiation and signaling. Many studies have confirmed genetic variants of *FAM167A/BLK* to be associated with systemic lupus erythematosus (SLE) as well as multiple other autoimmune diseases, including systemic sclerosis, rheumatoid arthritis and Sjögren's syndrome [12]–[19].

Here, we implement the PCA approach in our large lupus registry cohort (LFRR) at the 8p23 region in order to determine the influence of inversion on previously known association signals for lupus.

## Methods and Subjects

### Ethics statement

The detail of recruitment and biological sample collection of lupus cases and controls has been described in detail previously [20], [21]. Samples were supplied from multiple investigators from different institutions with approval from their respective institutional review boards (IRBs). All study participants provided written consent prior to study enrolment; consent forms were obtained at each location under IRB guidelines. Samples were then assembled at the Oklahoma Medical Research Foundation (OMRF) and the study protocols (including the enrollment process, consent forms, collection of DNA and subject information) for this study were approved by the Oklahoma Medical Research Foundation (OMRF) Institutional Review Board.

### Recruitment and Biological Sample Collection

Briefly, samples were obtained from the “Large Lupus Association Study #2” (LLAS2) in which 16,500 individuals were genotyped previously, including 8068 Caucasians as previously described [20], [21]. LLAS2 is a project investigating genetic associations in SLE using a candidate gene approach [20]. All SLE cases met the 1997 ACR classification criteria for SLE. Individual ethnicities were self-reported and genetic outliers were removed by principal component analysis. All genotype data were generated using the Illumina iSelect at the Oklahoma Medical Research Foundation (OMRF) genotyping facility as previously described [20], [21].

### Statistical Analyses

Principle component analysis (PCA) was performed using EIGENSTRAT and eigenvectors were generated using post-quality control data from 7416 Caucasian samples and 459 polymorphic typed markers that passed standard quality control measures ((MAF>0.01, genotyping rate>0.95% and HWE<0.0001) [22]. These markers reside in the inversion region of 8p23.1 (from 7.2 to 12.4 MB of chromosome 8). In addition 267 HapMap3 Caucasian samples including 165 CEU (Utah residents with Northern and Western European ancestry from the CEPH collection) and 102 TSI (Toscani in Italia)) were also merged into the study as a validation step. The eigenvalues from the first axis of principal component (PC1) were used for K-means clustering assuming three clusters (K = 3). Clustering was performed using the Matlab k-means algorithm, with squared Euclidean distance as the distance measurement (http://www.mathworks.com/help/stats/kmeans.html). SNP allelic association was evaluated between cases and controls using the ×2 test with 1 d.f. The allelic odds ratio (OR) and 95% confidence intervals (95% CIs) were calculated using PLINK [23]. Logistic regression and genotypic conditional analyses were also performed using PLINK, controlling for the effect of a specific SNP or inversion status. Haploview version 4.2 was used to estimate the linkage disequilibrium (LD) between markers and estimate haplotype frequency [24]. Golden-Helix version 8.5.1 was used to graphically display the association results (Golden Helix, Inc., Bozeman, MT, www.goldenhelix.com).

## Results

7416 homogenous post-quality control European samples from LFRR were used for this study. The demographic distribution of these samples is shown in table 1. PCA analysis was performed using markers inside the predicted inverted territory (from 7.2 to 12.4 MB of chromosome 8). Since some of the HapMap samples have been previously typed by a FISH assay in other studies, we first merged publicly available genotyping data of these HapMap samples into our cohorts (HapMap3_r3_b36_fwd.consensus, http://hapmap.ncbi.nlm.nih.gov/). In this region, 459 markers were available that passed standard quality control criteria (MAF>0.01, genotyping rate>0.95% and HWE<0.0001); 287 of these markers overlapped with hapmap-3 genotyping data. PCA analyses were performed on 7683 samples (7416 Europeans and 267 HapMap-CEU/TSI).

**Table 1:** Demographic distribution of the individuals in the study*.

	Caucasians
Total	3926/3490
Male	344/1151
Female	3582/2339

* Values are the number of patients/number of controls.

**Figure 1:**
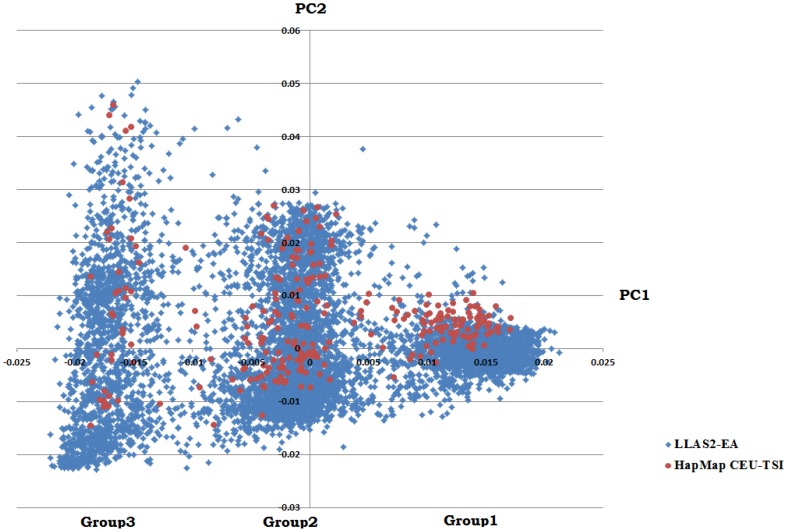
PCA analyses on 7683 Caucasian derived samples and identification of three subgroups at 8p23. Group 1 =  Inverted-homozygous, Group 2 =  Heterozygous, Group 3 =  Non-inverted homozygous.

**Figure 2:**
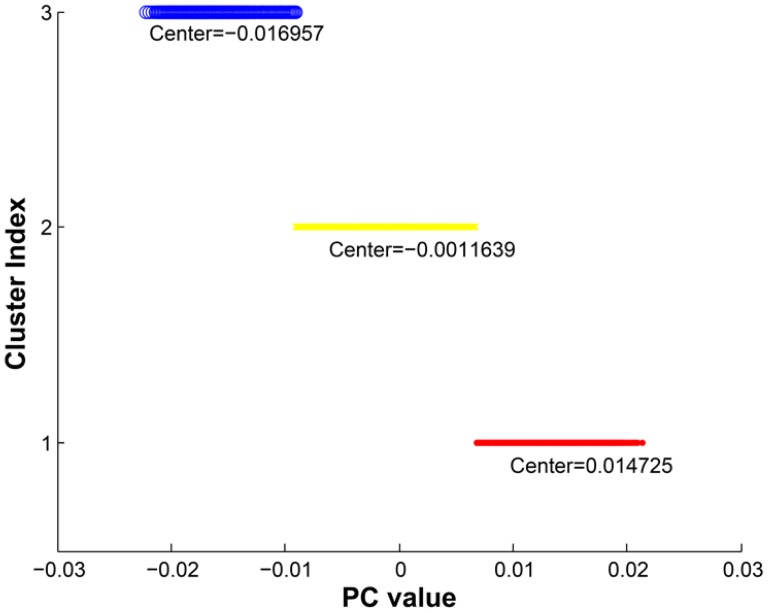
Cluster analyses using K-means clustering with squared Euclidean distance measurement. PC value is a numerical value obtained from STRUCTURE PC1 vector for each individual. In this approach the algorithm attempts to find the centers of the clusters in order to minimize the sum of the distances within each cluster.

As predicted, the PCA results identified 3 confined clusters consistent with previous analyses (Fig. 1), [6]. In order to better assign individuals to each cluster as recommended in a previous publication [6], we performed cluster analyses implementing the K-means clustering approach considering 3 clusters, and then assigned individuals into 3 subgroups as shown in Fig. 2. Although this method effectively clusters individuals into 3 groups, using PCA per se was not sufficient to determine which cluster is homozygous-inverted and which one is homozygous non-inverted. In order to address this, we merged the HapMap data set as described and then evaluated the concordance rate and the status of inversion according to FISH results available in the public domain. Indeed, our PCA methods were able to correctly call 59 out of 60 available HapMap samples with previous typed FISH results and assign them into 3 subgroups with a concordance rate of>98% [11], (S1 Table). These comparisons also determined which group on either side of the middle heterozygous cluster was inverted homozygous and which cluster belong to non-inverted homozygous. As shown in Fig. 1, we found that group 1 must be inverted homozygous and group 3 non-inverted homozygous. The overall frequency of each subgroup in our Caucasian population was 29% inverted homozygous, 49% heterozygous and 22% non-inverted homozygous. The inversion status was in HWE in our collection (p = 0.45). The ratio of lupus cases to controls in each subgroup was determined with the highest ratio belonging to non-inverted homozygous (1.36) (for inverted homozygous it was 0.97 (1067 cases and 1095 controls), for heterozygous group 1.12 (1935 cases 1717 controls) and non-inverted subgroups it was 1.36 (924 cases and 678 controls)) (Table 2).

**Table 2:** Association and comparisons of minor allele frequency of known SNP rs13277113 for lupus according to three identified Caucasian subgroups.

Clusters	Case/Controls	CHR	SNP	BP	Minor	case	control	Major	CHISQ	P	OR	L95	U95
Group1 = Inverted homozygous	1067/1095	8	rs13277113	11349186	A	0.0177	0.007	G	9.929	0.00163	2.456	1.379	4.375
Group2 = Heterozygous group	1935/1717	8	rs13277113	11349186	A	0.3035	0.27	G	7.635	0.00572	1.153	1.042	1.276
Group3 = Non-inverted homozygous	924/678	8	rs13277113	11349186	G	0.4139	0.47	A	11.04	0.00089	0.7861	0.682	0.9061

**Figure 3:**
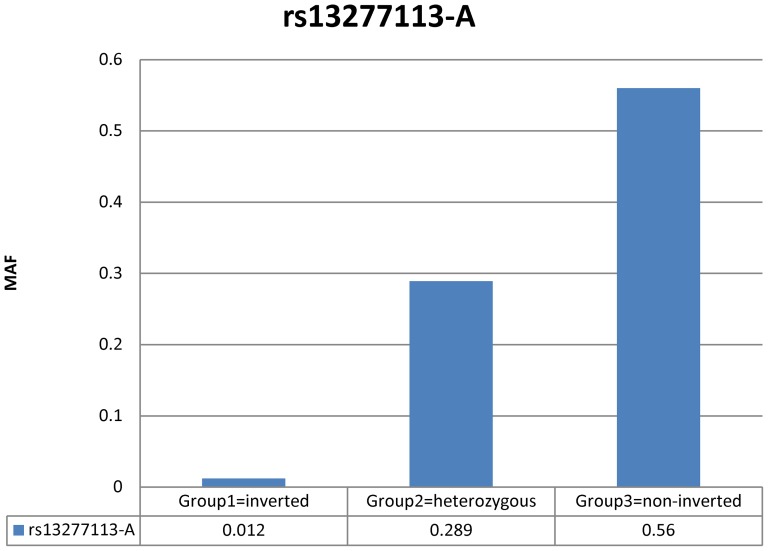
Comparisons of minor allele frequency differences of a known published SNP associated with lupus (rs13277113) in 3 different Caucasian subgroups.

In order to further confirm the results of the PCA analyses and to make sure that the estimate for subgrouping is correct, we compared the minor allele frequency of one of the published and common SNPs associated with lupus, rs13277113, to these three subgroups. Indeed, we observed significant minor allele frequency differences among the 3 groups that range from 1% in one group to a major allele frequency of 56% in another group (Fig. 3). These significant variations in allele frequency further confirm the PCA methods in defining subgroups. Interestingly, despite significant variation in allele frequency in different groups, the effects of the association of SNP rs13277113 with lupus were consistent across the three sub groups (Table 2).

We then performed association analyses conditioning on inversion status of individuals. Table 3 shows the summary of statistical results of top associated variants with previous results of p<10^-8^. We found that the association effects, although weaker, remain significant for most of the top lupus-related variants including rs13277113 and rs998683 (Table 3). The overall effect of all association results before and after controlling for inversion has been shown in table 2.

**Table 3:** The logistic regression conditional analyses and association results of selected top markers known to be associated with lupus.

CHR	SNP	BP	Minor	Case	Control	Major	P-original*	OR	L95	U95	P-conditional	OR-conditional	L95	U95	R2**
8	rs2409718	11012977	G	0.5133	0.4617	A	2.52E-09	1.229	1.151	1.313	0.00307	1.218	1.069	1.388	0.75
8	rs13257831	11332964	C	0.3344	0.2852	G	1.23E-09	1.259	1.173	1.352	0.0002279	1.187	1.084	1.301	0.39
8	rs2736340	11343973	A	0.302	0.2476	G	1.01E-12	1.315	1.222	1.415	1.36E-07	1.288	1.172	1.414	0.39
8	rs2618473	11344127	A	0.3088	0.2562	G	5.81E-12	1.297	1.207	1.394	2.77E-07	1.268	1.158	1.389	0.36
8	rs4840565	11345545	G	0.3294	0.2767	C	6.96E-11	1.284	1.195	1.379	7.13E-06	1.223	1.12	1.335	0.34
8	rs1478901	11347833	G	0.2904	0.2346	C	1.82E-12	1.335	1.236	1.442	2.33E-07	1.292	1.172	1.424	0.38
8	rs13277113	11349186	A	0.2901	0.2376	G	2.93E-12	1.311	1.217	1.413	2.15E-07	1.288	1.171	1.418	0.38
8	rs4840568	11351019	A	0.3237	0.271	G	4.05E-11	1.288	1.198	1.384	1.35E-06	1.24	1.137	1.354	0.32
8	rs922483	11351912	A	0.3413	0.2902	G	2.00E-10	1.267	1.182	1.359	2.71E-06	1.221	1.123	1.327	0.30
8	rs2736345	11352485	G	0.3538	0.2994	A	1.63E-11	1.281	1.196	1.373	5.30E-07	1.24	1.14	1.348	0.32
8	rs2618476	11352541	G	0.3094	0.2547	A	6.38E-13	1.311	1.22	1.409	5.95E-08	1.298	1.181	1.427	0.41
8	rs998683	11353000	A	0.3074	0.253	G	8.19E-13	1.31	1.218	1.409	5.92E-08	1.3	1.182	1.429	0.41
8	rs2249040	11390779	T	0.461	0.4087	A	2.76E-08	1.237	1.157	1.323	0.009028	1.134	1.032	1.247	0.58
8	rs9657551	11398183	G	0.3782	0.3298	A	2.20E-09	1.236	1.155	1.323	0.0001727	1.191	1.087	1.305	0.44
8	Inversion†	—	N	0.4818	0.4404	I	8.18E-07	1.18	1.10	1.26	—	—	—	—	—

*R2: correlation coefficient as a measure of linkage disequilibrium between inversion status and reported marker in Caucasian population.

**P-original =  the original case control association results adjusted for three ordinary principal components.

† Inversion as a genotype call. N = Non-inversion, I =  Inversion

Considering the inversion status as a genotype call (II, IN, NN), we then calculated the degree of LD between inversion status and top associated markers with lupus. Consistent with previous reports, no markers were detected with perfect proxy with inversion (r^2^ = 1). As shown in Table 3, markers with higher LD with inversion status such as rs2409718 (r^2^ = 0.75) or rs2249040 (r^2^ = 0.58) produced less significant association results with lupus after conditioning for inversion status (Table 3). Furthermore, we detected an association effect between inversion status and SLE in our cohorts (Table 3) in which non-inversion condition was more frequent in Caucasian lupus cases than controls (p = 8.18×10^−7^, OR = 1.18, 95%CI = 1.10–1.26). Further sub-phenotype analyses suggest some degree of improvement in size effect of association of N allele with lupus nephritis (OR = 1.27, 95%CI = 1.15–139, p = 1.47×10^−6^) or presence of thrombocytopenia (OR = 1.43, 95%CI = 1.23–1.65, p = 2.00×10^−6^).

**Figure 4:**
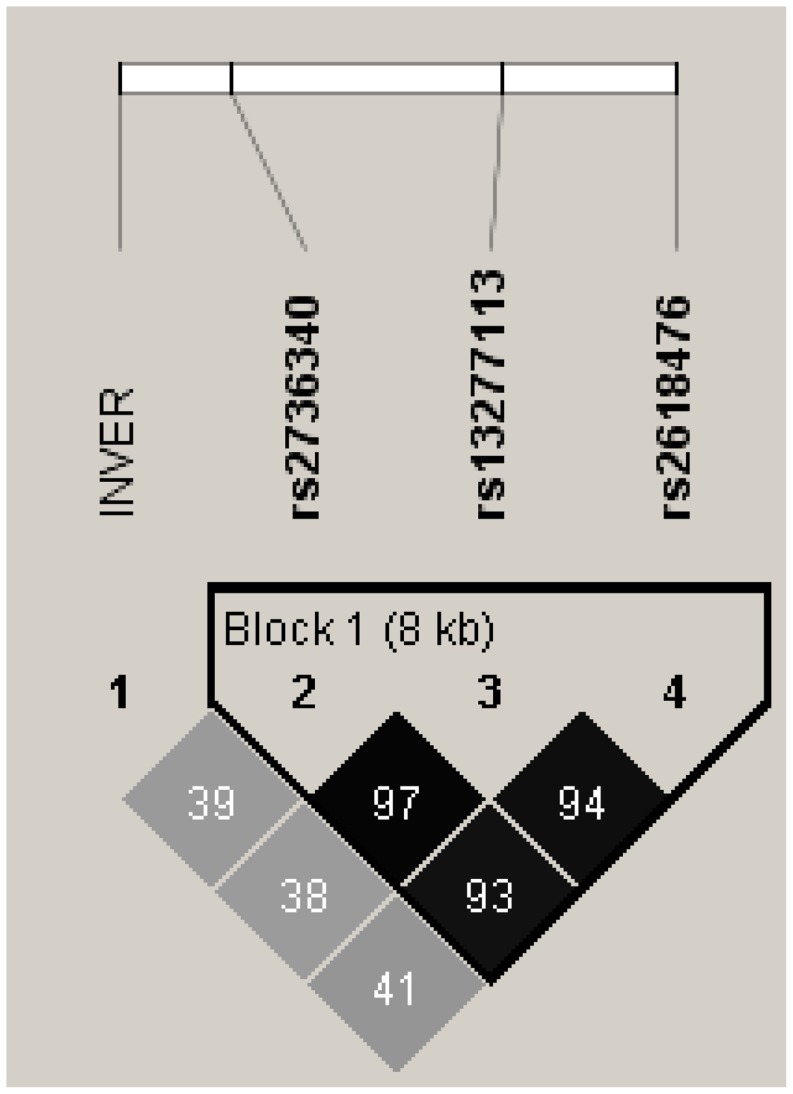
A schematic representation of haplotype block of 3 published lupus related snps at or near BLK and their degree of LD (r2) with inversion status (rs2736340, rs13277113, rs2618476). INVER = putative inversion variant.

In addition, haplotype analyses using three previously published lupus-associated SNPs (rs2736340, rs13277113, rs2618476) [25]–[27] revealed that the non-inversion status is indeed in LD with the AAG known risk haplotype of lupus risk. This risk haplotype (N-AAG) had a frequency of 29% in cases and 24% in controls (p = 4.57×10^−11^) (Table 4 and Fig. 4).

**Table 4:** The haplotype frequencies of the three known SNPs (rs2736340, rs13277113, rs2618476) and their relation with inversion status.

Block	Haplotype frequency	Case/Control Frequencies	P Value
I*-GGA	0.529	0.508/0.554	2.04E-08
*N-AAG*	0.266	0.288/0.240	4.57E-11
N-GGA	0.185	0.180/0.189	0.1521
N-GGG	0.01	0.011/0.009	0.223

I = Inverted, N = Non-inverted.

The non-inverted status is in LD with the known risk haplotype AAG (italics).

**Figure 5:**
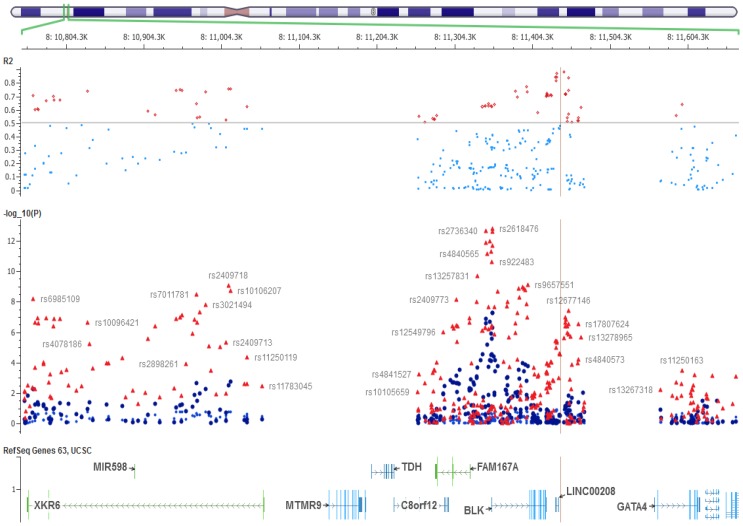
A schematic representation of the association map for Lupus at 8p23 before and after conditional analyses. Red dots =  original association, Blue dots = Association effects after conditioning on inversion. R2 =  correlation coefficient as a measure of linkage disequilibrium between inversion status and all markers in Caucasian population. The highest region in LD with inversion is shown with vertical line at LINC00208.

Finally, a region with highest LD with inversion (0.75>r^2^>0.85) was identified at or near long non coding RNA (LINC00208) distal to BLK locus (Fig. 5). Available markers in this region (Chr8:11.43–11.45 Mb) with r^2^>0.80 include rs10108511, rs2898290, rs2409798, rs10097870. This effect was confirmed in other European-derived data bases (data not shown).

## Discussion

In this study we validate the novel PCA methodology to identify inversion status in our large Caucasian cohort and evaluate this potential confounding factor in regard to the known *BLK* association with lupus. Indeed, in almost all HapMap samples (59 out of 60), our approach for determining inversion was in agreement with the experimental results by FISH reported in another study [11]. In addition, several of these HapMap samples were also previously typed in other independent studies and all were concordant with ours (see S1 Table) [3], [5], [28]. However, as reported previously, this methodology is not a reliable strategy for identification of inversion status in other ancestries when the suppression of recombination event between inverted and non-inverted chromosomal segments is only moderate and the initial inversion happened too long ago. The 8p23 inversion in the African population and the Asian population are such examples [5], [6].

One of the purposes of this study is to determine the potential confounding effect of inversion on a previously reported association with lupus at the *BLK* locus. Our data indicate that whether we perform association study in three identified subgroups independently (Table 2) or perform conditional analyses controlling for inversion background (Table 3), the association effect at the *BLK* region with lupus will remain significant (Table 2 & Fig. 4). However, in this study, we also found an association of non-inverted status with lupus as an additional risk factor for SLE in Caucasian (p = 8.18×10^−7^, OR = 1.18, 95%CI = 1.10–1.26). This novel result was consistent with a previous report in which the lupus risk haplotype was found to reside in non-inverted background in HapMap CEU samples (Table 4), [11]. Obviously part of this association is due to stratification bias of lupus cases because of LD and correlation between non-inverted status and lupus risk haplotype (Table 3&4). In any case, 8p23 inversion, similar to many inversions in humans, regulates gene expression of many genes in its territory as well as exerting indirect effects by maintaining allelic configurations [29]. Previous reports indicate that the number of N alleles (non-inversion status) is additively associated with decreased expression of *BLK* [11]. In addition, in the same report, 8p23 inversion is also robustly associated with expression of other genes in this territory including *XKR6, PPP1R3B, FAM167A, CTSB* and sometimes with opposite directions [11]. In a recent lupus functional study of BLK the risk allele (T) at rs922483 was shown to reduce proximal *BLK* promoter activity and modulated alternative promoter usage [30]. Allele T of SNP rs922483 is a known risk allele in LD with three above mentioned published variants and was correlated with non-inverted status in our analyses (r^2^ = 0.41) (Table 3). Therefore, both of these risk conditions i.e., non-inverted status (that we found to be more common in lupus cases) and risk allele-T synergistically tend to decrease expression of *BLK* in lupus patients. However, association of non-inversion status with lupus that alters regulation of multiple genes indicates that other genes in this region might also be important in pathogenesis of lupus perhaps in an orchestrated manner. In addition, the role of long non coding RNAs in the regulation of gene transcription should not be underestimated in which we found LINC00208 (C8orf14) had the highest LD with inversion status in different European derived populations.

In summary, our results add another dimension to the complexity of regulation of BLK in lupus and demand further studies to fully elucidate the interaction of inversion status and candidate functional variants.

## Supporting Information

S1 Table Predicted inversion calls for all 267 caucasian-derived population from HapMap CEU/TSI. *In Hapmap samples, Accession numbers beginning with NA refer to genomic DNA samples, while GM accessions refer to cell lines. ** 1 = Inverted homozygous, 2 = Heterozygotes, 3 = Non-inverted homozygous Red color: overlap samples that were in concordance with previous published FISH assay [11]. Additional confirmation: (a =  [5]; b =  [3]; c =  [28]). Yellow color: discordant call (called as Inverted in [11]).
(XLSX)
